# Carnitine Intake and Serum Levels Associate Positively with Postnatal Growth and Brain Size at Term in Very Preterm Infants

**DOI:** 10.3390/nu14224725

**Published:** 2022-11-09

**Authors:** Suvi Manninen, Sanna Silvennoinen, Paula Bendel, Maria Lankinen, Ursula S. Schwab, Ulla Sankilampi

**Affiliations:** 1 Institute of Public Health and Clinical Nutrition, University of Eastern Finland, 70211 Kuopio, Finland; 2 Department of Pediatrics, Kuopio University Hospital, 70210 Kuopio, Finland; 3 Department of Clinical Radiology, Kuopio University Hospital, 70210 Kuopio, Finland; 4 Institute of Clinical Medicine, Internal Medicine, Kuopio University Hospital, 70210 Kuopio, Finland; 5 Department of Pediatrics, University of Eastern Finland, 70210 Kuopio, Finland

**Keywords:** carnitine, acylcarnitine, preterm, postnatal growth, brain size, pediatric nutrition

## Abstract

Carnitine has an essential role in energy metabolism with possible neuroprotective effects. Very preterm (VPT, <32 gestation weeks) infants may be predisposed to carnitine deficiency during hospitalization. We studied the associations of carnitine intake and serum carnitine levels with growth and brain size at term equivalent age (TEA) in VPT infants. This prospective cohort study included 35 VTP infants admitted to Kuopio University Hospital, Finland. Daily nutrient intakes were registered at postnatal weeks (W) 1 and 5, and serum carnitine levels were determined at W1, W5, and TEA. The primary outcomes were weight, length, and head circumference Z-score change from birth to TEA, as well as brain size at TEA in magnetic resonance imaging. Carnitine intake at W1 and W5, obtained from enteral milk, correlated positively with serum carnitine levels. Both carnitine intake and serum levels at W1, W5, and TEA showed a positive correlation with weight, length, and head circumference Z-score change and with brain size at TEA. In linear models, independent positive associations of carnitine intake and serum carnitine levels with length and head circumference Z-score change and brain size at TEA were seen. In VPT infants, sufficient carnitine intake during hospitalization is necessary since it is associated with better postnatal growth and larger brain size at term age.

## 1. Introduction

Very preterm (VPT, born < 32 gestation weeks) infants are a vulnerable group of patients who have an increased risk for postnatal undernutrition, growth failure, and adverse neurodevelopmental outcome. At birth, nutritional supply via the umbilical cord is abruptly terminated, predisposing the VPT infant to a nutritional emergency. According to the current guidelines, parenteral nutrition (PN) should be started without delay, preferably in combination with minimal enteral feeding [1,2]. Transition to enteral feeding is, however, often delayed due to immature gut and associated morbidities requiring neonatal intensive care [2,3,4,5,6]. Postnatal growth failure is still common in infants born very preterm, and it is associated with adverse neurodevelopmental outcome [3,4,5,7,8,9].

The evidence informing many of the nutritional guidelines of VPT infants is scarce or controversial, and one nutrient whose role is not well understood in VPT nutrition is carnitine [1,2]. Carnitine is found in human milk and formulae, whereas it is typically completely lacking in PN solutions. According to current guidelines, carnitine supplementation is not uniformly recommended in PN for VPT infants since there are no data as to whether it promotes growth or health [10]. Carnitine has, however, an essential role in the energy metabolism by regulating the transportation of long-chain fatty acids into mitochondrial matrix [11]. Increasing evidence suggests that carnitine also has other biological roles, including neuroprotective effects in the developing brain [12].

During fetal life, the majority of the transplacental supply of carnitine from the mother occurs during the last trimester of pregnancy, when fetal growth is fast. Umbilical blood levels correlate with the birthweight of full-term newborns [13]. VPT infants miss out on the last trimester carnitine flux, and their tissue stores are low at birth [14]. They are at risk of developing carnitine deficiency due to limited tissue stores, immature endogenous synthesis, and insufficient intake from nutrition, with a concurrently increased need for carnitine due to rapid growth [11,14,15]. It is not known how circulating carnitine levels in VPT infants are associated with the nutritional intake or how the intake or serum levels of carnitine are related with postnatal weight, length gain, or brain growth.

In the present study, the associations of carnitine intake and serum carnitine levels with postnatal growth and brain size at term equivalent age were examined. Our hypothesis was, on the basis of the crucial role of carnitine in energy metabolism, that low carnitine intake and low serum carnitine levels are associated with each other, as well as with attenuated weight gain, linear growth, and brain growth in VPT infants.

## 2. Materials and Methods

As part of the Finnish PreBaby study on metabolism and growth in VPT infants, 35 infants (17 boys, 49%) were recruited during the first days of life for this prospective longitudinal study on the role of carnitine in nutrition at the Kuopio University Hospital, Finland, in 2009–2010 (Table 1). Their gestational age at birth varied from 23.4 to 31.9 weeks (median 26.7, SD 3.1), and the median birthweight was 900 g (range 475–1880, SD 410). There was one set of dichorial twins in the cohort. One infant died of necrotizing enterocolitis at the age of 49 days. The 34 infants who survived had typical morbidity associated with prematurity (Table 1). The median length of stay in the hospital was 67 days (range 28–182, SD 45.2 days), and the median postmenstrual age at discharge was 36.3 weeks.

Peripheral venous or arterial blood samples were drawn at weeks 1 (W1) and 5 (W5) postnatally and at TEA (median 40.0 postmenstrual weeks, range 37.7–42.0) for carnitine measurements. The serum samples were prepared by centrifugation, divided into aliquots, and stored at –70 °C until analyzed.

Parenteral and enteral nutrition (EN) practices adhered to current PN and EN guidelines [2,16]. The infants were given glucose and amino acid (Vaminolac^®^, Fresenius Kabi Ab, Uppsala, Sweden) infusions starting at birth, early (starting at day 1) lipid infusion (Clinoleic^®^ Baxter Oy, Helsinki, Finland), and trophic enteral feeding with their own mother’s milk or human bank milk. The amount of enteral nutrition was gradually increased according to tolerance to reach full enteral feeding as soon as possible. When daily enteral feeds exceeded 120 mL/kg, PN was discontinued.

Parenteral nutrition products (glucose infusions; Vaminolac^®^; Clinoleic^®^; parenteral vitamins; and trace elements Soluvit^®^, Vitalipid Infant^®^, and Peditrace^®^, Fresenius Kabi Ab, Uppsala, Sweden) did not include any carnitine, and additional carnitine supplementation was not provided. Maternal breast milk or pasteurized donor milk was fortified with Nutrilon BMF^®^ (Nutricia Medical, Turku, Finland) when the daily volume exceeded 100 mL/kg. If maternal breast milk was not available, preterm formula (Nutrilon Premilon^®^, Nutricia Medical, Turku, Finland) was introduced shortly prior to the discharge. At W1, none of the infants received formula, and at W5, four of them were on preterm formula.

At W1, W5, and TEA, nutrition was categorized as total parenteral, combined parenteral, and enteral or full enteral nutrition. Daily nutritional intake was registered during hospitalization. At TEA, most infants had been discharged and were feeding freely with fortified breast milk or formula at home. Average daily parenteral and enteral energy, fat, and protein intakes per weight (kg) were calculated at W1 and W5. Carnitine intake was calculated at W1 and W5 using estimated carnitine concentrations in breast milk (1.2 mg/100 mL) [22] and carnitine concentrations of formulas provided by the manufacturer. Nutritional intake at TEA could not be calculated in detail since most infants were feeding ad libitum.

Weight, recumbent length, and head circumference were registered at birth, and at W1, W5, and TEA (hospital or outpatient visit). Weight was measured using the Giraffe OmniBed inbed scale (Ohmeda Medical) in intensive care or a baby scale (Seca model 376, Seca, Hamburg, Germany) to the nearest 5 g, recumbent length using an infantometer (Holtain Ltd., Crymmych, Pembs, UK) to the nearest 0.1 cm, and the head circumference with a non-stretchable tape (Seca, Hamburg, Germany) as the maximum occipito-frontal diameter to the nearest 0.1 cm.

Brain magnetic resonance imaging (MRI) was performed without sedation during postprandial sleep as part of our hospital’s VPT clinical protocol using a 1.5T MRI system (Magnetom Avanto, Siemens, Erlangen, Germany) at TEA (median postmenstrual age 40.4 weeks, SD 1.0, range 37.9 to 42.2). According to the decision or the neonatologist, four VPT infants with gestational age at birth close to 32 weeks and normal brain ultrasound scans were not scanned. We used standard two-dimensional brain size metrics, which have been previously shown to correlate well with brain volume but are much easier to achieve than volumetric measurements [23]. Qualitative brain abnormalities were classified according to the degree of white matter and gray matter abnormality, and those with major brain injury were excluded from the brain metrics assessment (*n* = 3). The standardized brain metrics included bifrontal, biparietal, transverse cerebellar, and fronto-occipital diameters at standard locations on T2-weighted coronal MRI sequences [23]. The MRI images were scored individually by two neuroradiologists, and results were averaged. Intraclass correlation coefficient between the neuroradiologists varied from 0.75 to 0.98.

The study was carried out with approval of the Ethics Committee of the Pohjois-Savo Health District, Finland (decision number 11/2008). Informed consent was obtained from the parents at the recruitment of the infant.

### 2.1. Carnitine and Short-Chain Acylcarnitine Assays

Free carnitine and short-chain acylcarnitine concentrations in serum samples were measured using ultra-performance liquid chromatography–tandem mass spectrometry (UPLC-MS/MS) as described by Kivilompolo et al. [24]. For extraction, 10 µL of serum was mixed with 10 µL of labeled internal standard and 40 µL extraction solvent (acetonitrile–methanol, 3:1 (*v*/*v*)). After vortexing for two minutes, the samples were centrifugated for 5 min. The supernatant was removed and used for UPLC-MS/MS analysis. A Waters Acquity UPLC H-Class chromatograph was coupled to a Micromass Quattro micro triple quadropole mass spectrometer (Waters, Milford, MA, USA).

Chromatographic separation was performed on a Waters Acquity UPLC BEH Hilic (2.1 × 100 mm, 1.7 µm) column in gradient mode. Solvent A was 10 mM ammonium acetate with 0.2% formic acid in water, and solvent B was acetonitrile. The initial mobile phase composition was 10% solvent A and was linearly increased to 20% solvent A over 7 min. The total run time with column flush and re-equilibration was 10 min. The column temperature was 30 °C, and the flow rate was 0.5 mL/min.

Data acquisition was performed in positive electrospray ionization (ESI+), and the data were collected in the selected reaction monitoring (SRM) mode. The ion source parameters were as follows: source temperature, 125 °C; capillary voltage, 3 kV; desolvation gas temperature, 400 °C; desolvation gas flow, 800 L/h. Nitrogen and argon were used as cone and collision gases, respectively. The chromatographic conditions were optimized for the separation of free carnitine (L-carnitine, C0) and short-chain acylcarnitines (C2 and C3). Free carnitine and acylcarnitines are known to be mitochondrial biomarkers, and these carnitines were chosen to be measured in this study since they are known to have crucial roles in maintaining metabolic flexibility [25,26]. Metabolic flexibility refers to the capacity of the body to use different energy sources depending on the metabolic circumstances. Furthermore, short-chain acylcarnitines are derived from alternative energy sources and released into the circulation mainly by the liver [26,27].

### 2.2. Statistical Methods

Growth parameters (weight, length, and head circumference) were transformed into age- and sex-specific Z-scores using a contemporary population-based reference [17]. Small for gestational age (SGA) was defined as birthweight Z-score below −2 standard deviations [18]. The primary growth outcomes were the weight, length, and head circumference Z-score change between birth and TEA, as well as the brain metrics (bifrontal, biparietal, transverse cerebellar, and fronto-occipital diameters) at TEA.

The change of serum concentrations of carnitines as well as growth parameters by time were assessed using mixed linear models because of the potential correlation structure of the data caused by repeated measurements and twins. Sex, gestational age at birth, and time point at sampling (W1, W5, TEA) were modeled as fixed effects, and the subject and pair (twins) as random effects. Birthweight Z-score was included in the model as a covariate. Logarithmic transformations were performed for skewed variables. Normality of the residual distributions was assessed with histograms.

Several raw correlations were tested using Pearson’s correlation test or, in the case of skewed variables, Spearman’s rank correlation test. These included correlations between the parenteral or enteral energy intake with carnitine intake, correlations between the carnitine intake and growth parameters and brain metrics, as well as with the serum concentrations, and finally between the serum carnitine concentrations and growth parameters or brain metrics.

Linear regression models were constructed to study the associations between carnitine intake or serum carnitine levels with growth outcomes as well as brain size outcomes. The first model examined the possible associations of macronutrient intake (daily enteral energy (kcal/kg), parenteral energy (kcal/kg), fat (g/kg), protein (g/kg)), and mean carnitine intake during W1 and W5 (mg/kg), with the primary growth outcomes adjusted for gestational age at birth (Model 1). The second model tested the associations of nutritional intake with brain metrics at TEA and was adjusted for postmenstrual age at MRI scan (Model 2). The associations between the mean serum carnitine concentrations at W1, W5, and TEA, and the primary growth outcomes were further examined using a linear regression model adjusted for sex, birthweight Z-score, and gestational age at birth (Model 3). Finally, associations between the mean carnitine concentrations at W1, W5, and TEA and brain metrics at TEA were studied using a model adjusted for head circumference Z-score at birth and postmenstrual age (weeks) at the time of MRI scan (Model 4). *p*-values < 0.05 were considered statistically significant. Statistical analyses were performed using the IBM SPSS statistical software (v. 27, IBM Corp., Armonk, NY, USA).

## 3. Results

### 3.1. Postnatal Growth, Nutrition, and Carnitine Intake in VPT Infants

Median weight loss after birth was 6.3 percent (range 0–17 percent), and birthweight was regained at the median age of eight days (range 1–23 days). There was a significant drop in weight, length, and head circumference Z-score from −0.5, −0.5, and −1.0 at birth to −2.3, −2.7, and −2.5, respectively, at the age of 5 weeks (*p* < 0.001 for all) (Table 2). Catch-up growth started already before TEA, when weight, length, and head circumference Z-score had increased to −1.5, −1.4, and 0.1, respectively (W5–TEA pairwise comparison, *p* < 0.001 for all). However, weight and length Z-score at TEA remained lower than at birth (Table 2).

At W1 (median postmenstrual age 27.7 weeks), the majority of the infants had both enteral and parenteral nutrition (54%), and median daily energy intake during the first week of life was 71 kcal/kg (range 36–90). At W5 (median postmenstrual age 32.0 weeks), the majority of the infants (80%) were on enteral feeding, and median daily energy intake during the fifth week of life was 117 kcal/kg (68–143). Most of the infants on enteral feeding received fortified maternal milk until W5. At TEA, all the infants were on enteral feeding with either fortified maternal milk (*n* = 7) or preterm formula (*n* = 19), or both (*n* = 8).

**Figure 1:**
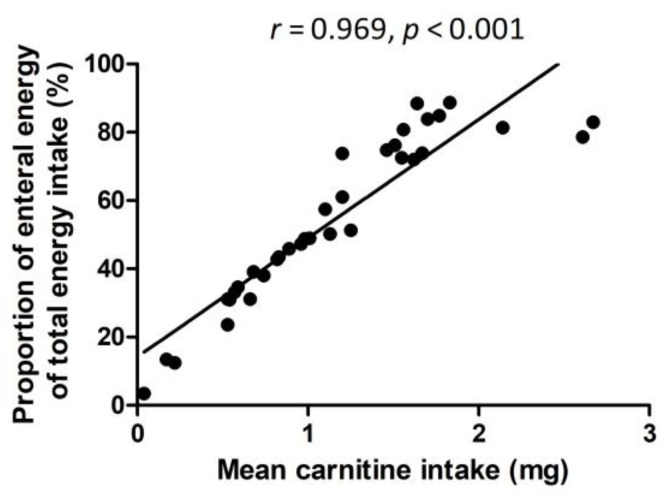
Spearman rank correlation between the mean daily carnitine intake (mg/kg) and proportion of mean enteral energy of the total daily energy (%) at weeks 1 and 5.

Parenteral nutrition did not include any carnitine, and therefore there was a significant positive correlation between the mean carnitine intake at W1 and W5 (mg/kg/day), with the proportion of mean enteral energy intake of the total energy intake at W1 and W5 (kcal/kg/day) (*r* = 0.969, *p* < 0.001) (Figure 1).

### 3.2. Associations of Carnitine Intake with Postnatal Growth and Brain Size at TEA

**Figure 2:**
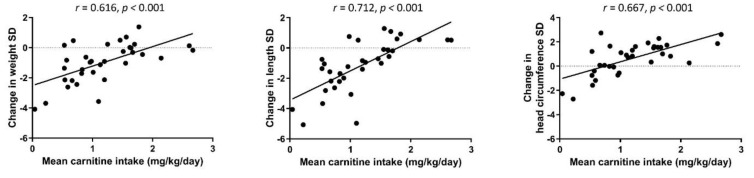
Spearman rank correlations between mean daily carnitine intake (mg/kg) of weeks 1 and 5, and weight, length, and head circumference Z-score change from birth to TEA.

There was a significant positive correlation between mean carnitine intake (W1 and W5) and change of weight, length, and head circumference Z-scores between birth and TEA (*r* = 0.616–0.712, *p* < 0.001 for all correlations), indicating that low carnitine intake correlated with a postnatal growth failure (Figure 2). Transcerebellar diameter at TEA also correlated with the mean carnitine intake (W1 and W5) (*r* = 0.442, *p* = 0.021).

Table 3 shows the results of the linear model on associations of the energy and macronutrient intake and carnitine intake (mean of W1 and W5) with the weight, length, or head circumference Z-score change from birth to TEA and brain size at TEA. Positive association of carnitine intake with both length and head circumference growth was observed (β = 0.480, *p* = 0.008; β = 0.425, *p* = 0.026, respectively). Other factors associated with better growth in weight, length, and head circumference were daily enteral energy and fat intake (β = 0.410–0.532, *p* < 0.05 for all), whereas parenteral energy intake was inversely associated with growth (Table 3). Intake of protein had no association with postnatal growth, except for length (β = 0.279, *p* = 0.049). Mean enteral energy intake and carnitine intake were positively associated and mean parenteral energy intake was inversely associated with transcerebellar diameter (β = 0.483, β = 0.666, and β= –0.514, respectively; *p* < 0.05 for all).

### 3.3. Postnatal Serum Carnitine Concentrations

**Figure 3:**
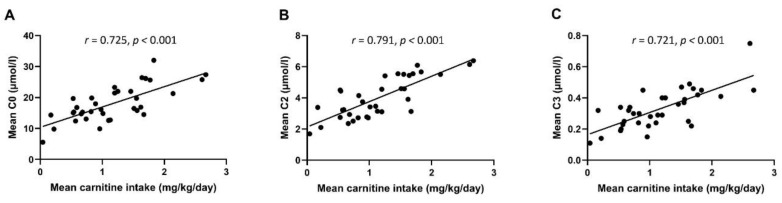
Pearson’s correlations between mean carnitine intake (mg/kg) of weeks 1 and 5 and the mean serum concentrations of free carnitine (C0) (**A**) and short-chain acylcarnitine C2 (**B**) and C3 (**C**) levels at week 1, week 5, and at term-equivalent age.

Serum concentrations of free carnitine and short-chain acylcarnitines increased from W1 to W5, and from W5 to TEA in a statistically significant manner (Figure S1, panels A–C, *p* < 0.05 for all comparisons). Carnitine intake (mean of W1 and W5) correlated significantly with the free carnitine as well as with short-chain acylcarnitine concentrations (*r* = 0.721–0.791, *p* < 0.001 for all, Figure 3).

### 3.4. Associations of Serum Carnitine Levels with Postnatal Growth and Brain Size at TEA

**Figure 4:**
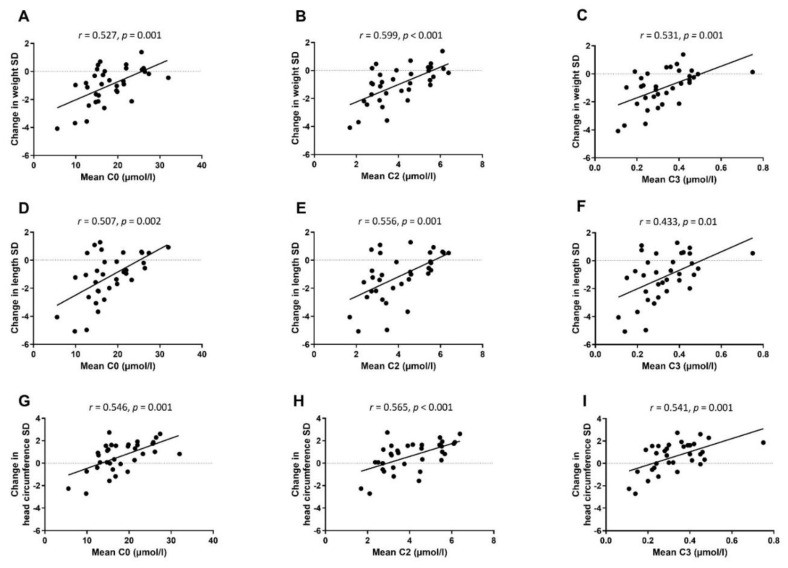
Spearman rank correlations between mean serum concentrations of free carnitine (C0) and short-chain acylcarnitines (C2 and C3) at week 1, week 5, and TEA, and weight (**A**–**C**), length (**D**–**F**), and head circumference (**G**–**I**) Z-score change from birth to TEA.

Correlations between mean concentrations of free carnitine and short-chain acylcarnitines C2 and C3 at W1, W5, and TEA, and postnatal growth are presented in Figure 4. Serum carnitine concentrations correlated positively with the better growth in weight (*r* = 0.527–0.599, *p* < 0.01 for all), in length (*r* = 0.433–0.556, *p* < 0.05 for all), and in head circumference (*r* = 0.541–0.565, *p* < 0.01 for all). In the linear regression model, associations remained significant between mean concentrations of short-chain acylcarnitines and weight Z-score change after adjusting for sex, birthweight Z-score, and gestational weeks at birth (Table 4).

Mean concentrations of free carnitine and short-chain acylcarnitine C3 (at W1, W5, and TEA) correlated positively with transcerebellar diameter (*r* = 0.566, *p* = 0.002; *r* = 0.447, *p* = 0.019, respectively). Furthermore, the mean concentration of free carnitine correlated with biparietal diameter (*r* = 0.386, *p* = 0.046). In the linear regression models adjusted for head circumference Z-score and postmenstrual age at the time of MRI, mean concentrations of free carnitine and short-chain acylcarnitines were positively associated with fronto-occipital and transcerebellar diameters (Table 4). Furthermore, mean concentration of free carnitine was positively associated with biparietal diameter, and mean concentration of acylcarnitine C2 was positively associated with bifrontal diameter.

## 4. Discussion

In the present study, we found that carnitine intake during the first 5 weeks of life in very preterm infants was dependent on the enteral intake of milk, and that free carnitine or short-chain acylcarnitine levels in serum reflected the carnitine intake. Higher carnitine intake during the first 5 weeks of life, as well as higher serum carnitine levels, were associated with better postnatal growth and larger brain size at TEA. Taken together, our findings highlight the importance of enteral feeding as a source of carnitine and the significance of carnitine intake for postnatal growth and brain growth in preterm infants.

Although human milk and infant formulas contain variable amounts of carnitine, previous evidence does not support routine use of parenteral carnitine supplementation for preterm infants [28]. The evidence available regarding carnitine supplementation is, however, controversial. Several supplementation studies have found that despite the higher circulating carnitine levels after parenteral carnitine supplementation, the clinical effect in terms of growth or hospital stay has been modest. Ramaswamy et al. [29] found in their retrospective cohort study that less than 10% of very preterm infants develop secondary carnitine deficiency, and concluded that prophylactic carnitine supplementation is not necessary for all preterm infants in total parenteral nutrition. Furthermore, in a prospective, double-blind, placebo-controlled study, very low birthweight preterm infants were given carnitine supplements first with 15 mg/kg/day intravenous infusions and after establishment of full enteral feeds, 100 mg/kg/d of carnitine orally in four divided doses [30]. Carnitine supplementation was not found to improve growth in that study. Similarly, in the studies of Shortland et al. [31] and Pande et al. [32], intravenous carnitine (25 mg/kg/day [31] or 8 mg/kg/day [32]) was not found to have an effect on postnatal growth in preterm infants in randomized controlled trials. In fact, current guidelines on neonatal parenteral nutrition state that carnitine supplementation may be considered in preterm infants on parenteral nutrition for more than 4 weeks or on the basis of individual assessment [33]. However, it is not known as to whether carnitine supplementation could be beneficial during prolonged parenteral nutrition (>1 month) [28].

In the present study, we adhered to existing recommendations and did not provide carnitine supplementation during parenteral nutrition. The only external source of carnitine was the enteral milk. To our knowledge, this is the first study to investigate the effects of carnitine intake from milk feeding on growth and brain size and on serum carnitine concentrations in a longitudinal study design. In opposition to the previous studies assessing effects of carnitine supplementation in preterm infants, we showed that higher carnitine intake during the first 5 weeks of life was associated with better postnatal growth and larger cerebellar size at TEA. Existing evidence supports human milk feeding in preterm infants, although it has been associated with slower weight gain [34] and lower plasma carnitine levels [35] than infant formula feeding. As reviewed recently by Ottolini et al. [36], macronutrient, micronutrient, and energy contents of preterm human milk vary significantly, and the individualized fortification of human milk improves both growth and neurodevelopmental outcome of preterm infants. It remains to be studied whether carnitine levels of preterm maternal milk also vary, as well as whether individualized supplementation of human milk according to carnitine contents would be beneficial.

In addition to dietary intake, circulating carnitine levels reflect the endogenous synthesis and renal absorption of carnitine. However, circulating carnitine levels may not reflect tissue carnitine stores. Preterm infants are at risk of developing carnitine deficiency due to immature endogenous synthesis, limited tissue stores, and insufficient exogenous intake with concurrently increased need due to rapid growth [11,14,15]. Studies investigating carnitine levels of preterm newborns have shown inconsistent findings [13,35,37,38,39,40,41,42]. Gestational age and birthweight have been shown to play a significant role in the differences in blood carnitine profiles of preterm infants, indicating that tissue stores are limited, and endogenous synthesis is immature [43,44,45]. Clark et al. [10] showed a decline in free carnitine in preterm infants on total parenteral nutrition without carnitine supplementation, indicating dependence on exogeneous carnitine supply. Our results showed that free and short-chain acylcarnitine concentrations in serum can be regarded as reliable markers of carnitine intake. Furthermore, our results showed that serum carnitine levels were very low during the first weeks of life. These results are in line with the observations of Clark et al. [10] and support the view that endogenous carnitine synthesis is immature during the first weeks of life in very preterm infants.

To our knowledge, there are no previous studies investigating the associations between serum carnitine concentrations and postnatal growth of preterm infants in a longitudinal study design. Levels of acylcarnitine metabolites including medium-chain acylcarnitines (e.g., C5-OH, C8, and C12) have been previously found to be associated with postnatal growth failure and long-chain acylcarnitines (e.g., C18:1 and C18:2) with appropriate growth in extremely preterm infants [46]. In the present study, concentrations of short-chain acylcarnitines (C2 and C3) were associated with change in weight Z-score in a linear regression model.

To investigate the associations of carnitine intake and serum levels with brain size of very preterm infants at TEA, we used the TEA brain MRI scans taken during the normal clinical postnatal follow-up of very preterm infants. They were interpreted by two pediatric neuroradiologists independently, and two-dimensional brain metrics were used. This method is much easier to apply than volumetric measurements; a correlation between brain volume and linear measurements was previously shown, and its reproducibility is good [23]. Furthermore, it has been previously shown that these simple brain metrics at TEA MRI scan are reliable surrogates of neurodevelopmental outcome in VPT infants [47]. We showed that both carnitine intake during the first weeks of life and serum carnitine levels in VPT infants were independently associated with brain size in TEA MRI scan. These associations were independent of energy and macronutrient intakes, which could indicate that free and short-chain acylcarnitines may have an independent role in brain growth. Weeks 25 to 37 of gestation are a critical time of brain development, and preterm infants are vulnerable for impaired brain maturation and the rapidly growing brain for nutrient insufficiencies [48,49]. Carnitine and acylcarnitines are known to have several neuroprotective functions [12,50]. L-carnitine, or free carnitine, and its acetylated derivative acetyl-L-carnitine have potential to attenuate inflammation and prevent oxidative damage, but so far, these neuroprotective mechanisms and effects of L-carnitine or acetyl-L-carnitine have been studied only in preclinical models [12,49]. In addition, short-chain acylcarnitine C2 has been found to be essential in the biosynthesis of some neurotransmitters, such as acetylcholine [50], and in animal models, C2 has been found to modulate energy metabolism in the developing brain [51].

Our findings that carnitine intake as well as circulating carnitine levels were most strongly associated with cerebellar size at TEA were of special interest. Previous studies have revealed the importance of human milk feeding to regional brain volumes and the cerebellum in preterm infants [52]. Improved early brain growth has been found in preterm infants fed with breast milk as compared with formula-fed infants [53]. In addition to L-carnitine found in breast milk, among other components, lactoferrin has also been suggested to have neuroprotective potential [49,52]. Growth of the cerebellum is closely associated with neurodevelopment [54]. A reduced cerebellar size at TEA has been previously associated with significantly lower mental developmental indices, as well as a reduced biparietal diameter with significantly lower mental and psychomotor developmental indices in Bayley Scales of Infant Development at 2 years of age [55]. Further studies are needed to elucidate the role of carnitine in brain development of preterm infants.

A limitation of this study is the small study population and the lack of data on exact carnitine intake, especially after 5 weeks of life, as well as a lack of data on neurodevelopmental outcomes. Moreover, only 1.5 T brain MRI scans were available, and volumetric data on brain size were not stored. However, the strength of this study is the careful longitudinal follow-up of the infants and the use of detailed nutritional data during the first 5 weeks of life.

In conclusion, sufficient carnitine intake during hospitalization of very preterm infants is necessary since it is associated with better postnatal growth and larger brain size at term equivalent age. While there is no consensus on parenteral carnitine supplementation in VPT infants, these results strongly support the importance of early enteral feeding (preferably with fresh maternal milk) as soon as possible after birth in order to guarantee carnitine intake. Further studies are needed to elucidate the role of carnitine intake in brain growth and the development of very preterm infants. Finally, new randomized studies are needed to elucidate whether carnitine supplementation during parenteral nutrition, or carnitine fortification of enteral milk feeding, would improve brain growth and neurodevelopment in these high-risk infants.

## Figures and Tables

**Table 1:** Clinical characteristics of the 35 very preterm infants.

	Median/*n*	Range/%
Maternal age (years)	30.3	22.1–38.0
Cesarean section (*n*)	27	77
Twins (*n*)	2	6
Males (*n*)	17	49
At birth		
Gestational age, weeks	26.7	23.4–31.9
Weight, g	900	480–1880
Weight, Z-score ^a^	−0.5	−3.4–1.8
Length, cm	34.5	28.0–44.0
Length, Z-score ^a^	−0.5	−4.6–3.8
Head circumference, cm	23.5	19.4–29.5
Head circumference, z-score ^a^	−1.0	−3.6–1.2
Small for gestational age ^b^	6	17
Apgar 5 min	7	1–9
Morbidity		
Early or late onset sepsis	8	22.9
Necrotizing enterocolitis ^c^	3	8.6
Intraventricular hemorrhage grade 3–4 ^d^	3	8.6
Bronchopulmonary dysplasia ^e^	12	35.3
Retinopathy of prematurity	2	5.9
Exitus	1	2.9

^a^ Birthweight, length, and head circumference were converted to Z-score using the population-based birth size reference for singletons or twins and adjusted for parity [17]. ^b^ Small for gestational age was defined as birthweight or length Z-score below −2 standard deviation units [18]. ^c^ Necrotizing enterocolitis was defined as Bell stage II or III [19]. ^d^ Intraventricular hemorrhage was diagnosed with ultrasonography using Papile’s classification [20]. ^e^ Bronchopulmonary dysplasia was diagnosed at 36 postmenstrual weeks using the oxygen reduction test [21].

**Table 2:** Nutrition, carnitine intake, and longitudinal growth from birth to term equivalent age (TEA) and brain size at TEA in 35 very preterm infants.

	At Birth		Week 1		Week 5		TEA ^a^	
	Median	Range	Median/*n*	Range/%	Median/*n*	Range/%	Median	Range
Gestational/postmenstrual age, weeks	26.7	23.4–31.9	27.7	24.4–33.0	32.0	28.4–36.9	40.0	37.7–42.0
Full enteral feeding (*n*, %)			9	25.7	28	80.0	34	100
Combined parenteral and enteral (*n*, %)			19	54.3	4	11.4	0	0
Total parenteral nutrition (*n*, %)			7	20.0	3	8.6	0	0
Daily enteral energy intake, kcal/kg			15	0–66	102	1–142		
Daily parenteral energy intake, kcal/kg			53	1–142	9	0–87		
Daily energy intake, kcal/kg			71	36–90	117	68–143		
Daily protein intake, g/kg			2.2	1.5–3.1	3.3	2.5–3.8		
Daily fat intake, g/kg			1.9	0.6–4.0	6.3	1.8–8.3		
Daily carnitine intake mg/kg			0.3	0–1.2	1.9	0–4.4		
Weight, g	900	480–1880	900	520–1810	1330	730–2500	2965	2000–4020
Length, cm	34.5	28.0–44.0	35.0	28.5–44.7	38.7	31.7–47.5	47.8	42.5–53.4
Head circumference, cm	23.5	19.4–29.5	23.5	19.9–30.1	27.4	22.0–34.1	35.3	31.9–38.0
Weight, Z-score ^b^	−0.5	−3.4–1.8	−1.6	−4.1–1.0	−2.3	−4.2–−0.3	−1.5	−3.8–0.77
Length, z_score ^b^	−0.5	−4.6–3.8	−1.2	−4.3–2.9	−2.7	−5.5–−0.3	−1.4	−4.9–1.4
Head circumference, Z-score ^b^	−1.0	−3.6–1.2	−1.7	−3.5–0.5	−2.5	−4.2–0.5	0.1	−2.4–1.9
Change in growth Z-score from birth								
Weight Z-score change			−1.1	−2.4–0.4	−1.7	−3.0–−0.2	−0.9	−4.1–1.4
Length Z-score change			−0.7	−1.3–0.6	−1.0	−4.5–1.1	−1.0	−5.1–1.3
Head circumference Z-score change			−0.9	−1.6–0.4	−1.1	−4.4–1.2	0.9	−2.7–2.7
Brain magnetic imaging at TEA ^c^ (*n* = 27)								
Bifrontal diameter, mm							65.4	59.3–72.5
Biparietal diameter, mm							75.1	66.3–80.0
Transcerebellar diameter, mm							51.5	46.5–55.5
Fronto-occipital diameter, mm ^d^							114.8	104.4–120.3

^a^ Nutritional intake at TEA was not available since most infants were discharged from the hospital and feeding freely with breast milk or formula. ^b^ Weight, length, and head circumference were converted to age- and sex-specific Z-score using the population-based birth size reference for singletons and intended for growth follow-up in preterm infants from birth to term [17]. ^c^ The simple brain metrics were measured at standard locations on T2-weighted coronal MRI sequences according to the method described by Nguyen The Tich et al. [23]. Thirty infants were scanned, and three were excluded due to major lesions in brain tissue. ^d^ *n* = 26.

**Table 3:** Associations of daily enteral and parenteral energy, macronutrient, and carnitine intakes (mean of weeks 1 and 5) with postnatal weight, length, and head circumference Z-score change from birth to term equivalent age (TEA) and brain size at TEA (linear regression model).

	Weight Z-Score Change		Length Z-Score Change		Head Circumference Z-Score Change		Biparietal Diameter (mm)		Bifrontal Diameter (mm)		Transcerebellar Diameter (mm)		Fronto-Occipital Diameter (mm)	
Daily Intake	β ^a^	*p*-Value	β ^a^	*p*-Value	β ^a^	*p*-Value	β ^b^	*p*-Value	β ^b^	*p*-Value	β ^b^	*p*-Value	β ^b^	*p*-Value
Enteral energy, kcal/kg	0.443	**0.010**	0.532	**<0.001**	0.499	**0.003**	0.135	0.544	0.313	0.154	0.483	**0.022**	0.105	0.640
Parenteral energy, kcal/kg	−0.336	**0.048**	−0.457	**0.008**	−0.490	**0.005**	−0.182	0.491	−0.320	0.150	−0.514	**0.016**	−0.132	0.564
Fat, g/kg	0.410	**0.014**	0.490	**0.002**	0.420	**0.011**	0.038	0.863	0.241	0.272	0.417	0.051	0.045	0.842
Protein, g/kg	0.267	0.075	0.279	**0.049**	0.131	0.381	0.117	0.571	0.060	0.774	−0.019	0.927	0.061	0.774
Carnitine, mg/kg	0.342	0.082	0.480	**0.008**	0.425	**0.026**	0.253	0.284	0.364	0.120	0.666	**0.002**	0.232	0.335

^a^ Linear regression model adjusted for gestational age at birth, standardized coefficients. ^b^ Linear regression model adjusted postmenstrual age at the time of MRI scan, standardized coefficients. Statistically significant *p*-values are highlighted in bold.

**Table 4:** Associations between mean serum carnitine concentrations (week 1, week 5, and term equivalent age (TEA)) and postnatal weight, length, and head circumference change from birth to TEA (*n* = 34) and two-dimensional brain diameters at TEA (*n* = 27) in a linear regression model.

	Weight Z-Score Change		Length Z-Score Change		Head Circumference Z-Score Change		Biparietal Diameter (mm)		Bifrontal Diameter (mm)		Transcerebellar Diameter (mm)		Fronto-Occipital Diameter (mm)	
Serum Carnitine Concentration	β ^a^	*p-*Value	β ^a^	*p-*Value	β ^a^	*p-*Value	β ^b^	*p-*Value	β ^b^	*p-*Value	β ^b^	*p-*Value	β ^b^	*p-*Value
Free carnitine	0.244	0.078	0.168	0.300	0.230	0.216	0.397	**0.026**	0.337	0.092	0.611	**<0.001**	0.350	**0.028**
Short-chain acylcarnitine C2	0.298	**0.032**	0.081	0.630	0.175	0.365	0.364	0.061	0.433	**0.040**	0.452	**0.028**	0.370	**0.031**
Short-chain acylcarnitine C3	0.255	**0.032**	0.149	0.297	0.282	0.082	0.243	0.202	0.232	0.267	0.543	**0.004**	0.334	**0.042**

^a^ Linear regression model was adjusted for sex, birthweight Z-score, and gestational weeks at birth, standardized coefficients. ^b^ Linear regression model was adjusted for head circumference Z-score at birth and postmenstrual age at the time of MRI scan. Statistically significant *p*-values are highlighted in bold.

## Data Availability

The data that support the findings of this study are not publicly available due to their containing information that could compromise the privacy of research participants.

## Supplementary Materials

The following supporting information can be downloaded at: https://www.mdpi.com/article/10.3390/nu14224725/s1, Figure S1. Serum concentrations of free carnitine (A) and short-chain acylcarnitine (B and C) levels at postnatal week 1, postnatal week 5, and term-equivalent age (TEA) in very preterm infants. Changes over time were tested using a linear mixed model, * *p* < 0.05.
