# Supplementary material for: Carnitine Intake and Serum Levels Associate Positively with Postnatal Growth and Brain Size at Term in Very Preterm Infants
Source: Nutrients. 2022 Nov 9;14(22):4725. doi: 10.3390/nu14224725 (PMC9696952; doi:10.3390/nu14224725)
Supplement: Supplementary file 1 [file nutrients-14-04725-s001.zip › nutrients-1997596-supplementary.pdf]

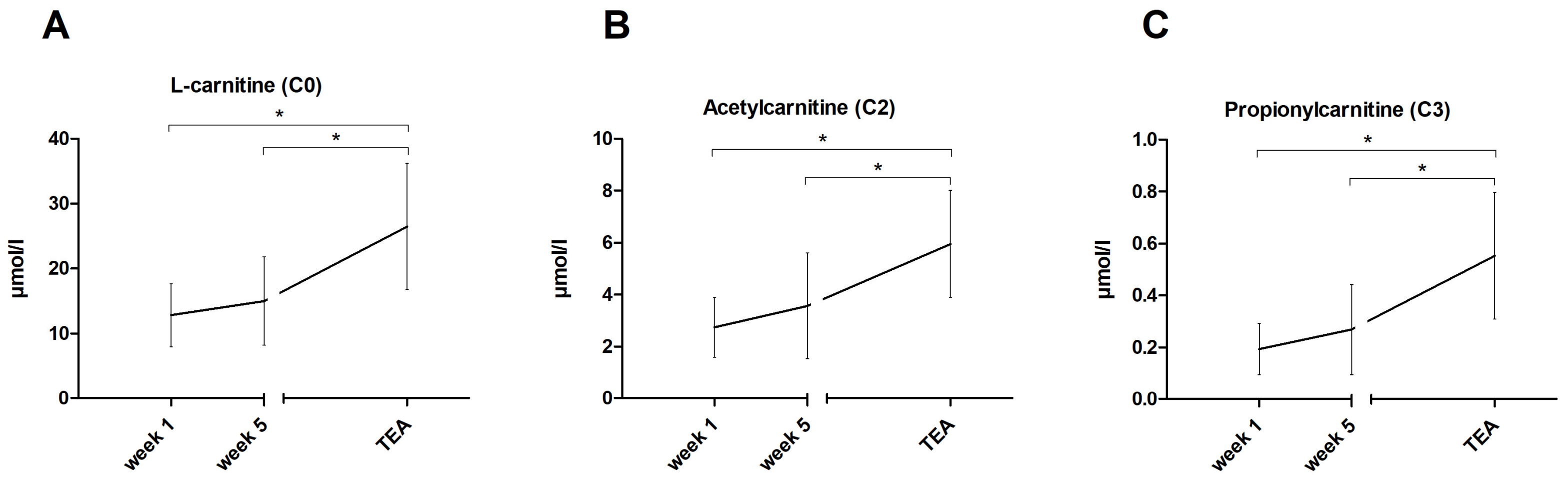


Figure S1. Serum concentrations of free carnitine (A) and short-chain acylcarnitine (B and C) levels at postnatal week 1, postnatal week 5, and term-equivalent age (TEA) in very preterm infants. Changes over time were tested using a linear mixed model, * $p < 0.05$.
